# Rheology, Strength, and Durability of Concrete and Mortar Made of Recycled Calcium Silicate Masonry

**DOI:** 10.3390/ma17122790

**Published:** 2024-06-07

**Authors:** Tanel Tuisk, Simo Ilomets, Tiina Hain, Joosep Kalbus, Targo Kalamees

**Affiliations:** Department of Civil Engineering and Architecture, Tallinn University of Technology, Ehitajate Tee 5, 19086 Tallinn, Estonia

**Keywords:** recycled building materials, calcium silicate brick, concrete aggregate, mortar aggregate, frost resistance

## Abstract

Selective demolition of building components and recycling construction demolition waste is a growing tendency as we move towards a circular construction. This study investigates the feasibility of using demolition waste from calcium silicate brick masonry as an aggregate in concrete and mortar. The purpose is to assess its impact on concrete and mortar properties, including compressive strength, durability, and workability. Silicate bricks from two demolished buildings were processed into aggregate, and laboratory experiments were conducted to evaluate concrete and mortar made with varying proportions of recycled aggregate. Results indicate that replacing natural aggregate (limestone rubble and sand) with recycled silicate brick aggregate up to 50% does not significantly compromise concrete performance, with no significant decrease in compressive strength observed. Frost resistance of the concrete made with recycled aggregate even surpasses that of reference concrete, possibly due to the lower density and higher (closed) porosity of the recycled aggregate. However, challenges such as increased water demand and loss of workability over time are noted with higher proportions of recycled aggregate. Further research is recommended to explore strategies for mitigating these challenges and to assess the effects of chemical admixtures on concrete properties. Overall, the findings suggest that recycled calcium silicate brick holds promise as a sustainable alternative for aggregate in concrete production.

## 1. Introduction

### 1.1. Background

Construction product regulation [1] sets basic requirements for construction. The sixth requirement in “Energy economy and heat retention” states that a building’s construction works must also be energy-efficient, using as little energy as possible during their construction and dismantling. The Energy Performance of Buildings Directive recast [2] sets ambitious goals for the building sector—to reach a “highly energy efficient and decarbonized building stock” by year 2050. As about 75–80% of today’s buildings will still be used in 2050 and today 75% of them are energy inefficient, deep renovation of existing building stock is needed. Deep renovation of existing building stock can support savings of 60–80% in thermal energy use. It is necessary and rational to renovate these buildings that will be used in the future. The removal of buildings from service may be driven by several reasons. The service life of a building may end because of economical, functional, esthetical, technological, legal, etc. reasons or because the building no longer fulfils basic requirements. A typical solution for a building after the end of service life is demolition.

Construction and demolition waste (CDW) are estimated to make up 33% of total waste generated annually in the EU [3]. About 85% of the CDW is mainly downcycled into backfill for careers, building sites, roads, building foundations, or new residential areas and industrial estates. Only about 3% of demolition waste is upcycled as reused/recycled material for the actual construction of new buildings [4]. Although a combination of bricks and ceramic tiles make up a large proportion of CDW, their use as aggregate may currently be prohibited, and these are typically landfilled [5]. To minimise CDW, it is necessary to ensure that mineral waste material undergoes preparation for reuse, recycling, or other recovery operations [6].

In addition to minimising the environmental impact, the use of CDW has also been motivated by the interruption of supply chains in recent years (Covid pandemic, wars) [7]. Since we live in limited resource conditions, the available material must be used very sparingly. To design building materials or buildings from reused, recycled, or selectively demolished elements, it is necessary to know the properties of materials and components. Batayneh et al. [8] recommend that concrete with recycled materials be used primarily in non-structural applications, where lower strength is required. Topçu and Sengel [9] and Folino and Xargay [10] showed that using CDW might reduce the workability, strength, and durability of concrete. Ruiz-Herrero [11] showed that concrete and mortar cellular materials containing plastic waste had weaker mechanical properties, and to improve them, the cement content had to be increased.

In many European countries, a large number of buildings were built in the years just after World War II, and today are at the end of their designed service life. The poor indoor climate [12] and large energy use [13] of these buildings also requires major renovation. It is reasonable to renovate those buildings that will remain in use after renovation for the next 50 years. Urbanisation leads to the restructuring of the location of populations—from rural areas to cities. Therefore, some buildings fall out of use [14], despite their technical condition, and materials from demolished buildings should be reused or recycled in the most effective way.

Today and in the future, the renewal of the built environment is moving towards circularity via (following the waste hierarchy):

- Selective demolition;
- Reuse of building components (low rate of reprocessing, maintaining the value);
- Circularity of building materials (average rate of reprocessing, low added value);
- New building materials by using secondary raw material (high rate of reprocessing, high added value);
- Smaller need for primary raw materials;
- Smaller amounts of CDW that need final disposal.

Derived from the aforementioned, both existing, as well as designed buildings, should be seen as material banks, where building materials have been placed temporarily. Until today, reuse and circularity in construction has been restricted because of legislation and the complexity of creating documentation for new products made of recycled materials.

### 1.2. The Need for Research

The properties and composition of CDW vary in different countries and depend on building traditions and local materials, including the age and service conditions of a material. The production of materials or products from CDW are not as universal as from primary raw materials, and there is higher uncertainty about the properties and quality of secondary raw materials. Different methods to characterize materials properties and quality of recycled CDW are available. Water content as one of key parameters for aggregate was determined also for calcium silicate brick, among other recycled aggregates [15].

Therefore, material properties and performance of secondary raw materials as well as new materials and products must be thoroughly studied. During the literature review, the authors of this paper found earlier studies where different recycled aggregates for concrete and mortars have been studied, e.g., concrete, mortar, ceramic brick, and tiles [16]. Thorough review covering numerous use of recycled fine aggregates in a form of table is given in [17]. In the Scopus database, there were 4005 results for “recycled aggregate concrete” AND “concrete”; 146 results for “recycled aggregate concrete” AND “brick”; 59 results for “recycled aggregate concrete” AND “stone”. However, we did not find any previous publications about concrete, where fine or coarse aggregates had been replaced with recycled crushed calcium silicate brick masonry—therefore, it was carried out in this paper to create new knowledge and to discover alternative applications. Practical outcomes would be decreasing the need for primary raw materials as well as producing fewer CO_2_ emissions from transportation if a “donor” building is located near the construction site of a new concrete building. As elsewhere, the approach could be applied in post-war countries and regions such as Ukraine or Israel and post-disaster Turkiye and Syria.

## 2. Materials and Methods

### 2.1. Donor Buildings for Demolition Waste

Two calcium silicate masonry buildings (“Ki”, “Ku”) were selected for the study.

**Figure 1:**
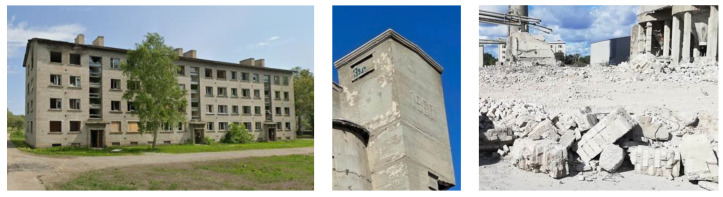
Donor buildings for demolition waste: the brick from “Ki” building (**left**) was used to produce concrete, the silicate sand used for the mortars brick from “Ku” building (**middle**, **right**).

“Ki” was a typical apartment building built in 1962 (coordinates 59.35656, 26.98132, ca 8 km south of the Gulf of Finland), Figure 1 (left). The building was located in a shrinking region—therefore, the building was abandoned approximately one year before the studies and slated for demolition. The building has four stories with three staircases and a full basement. All load-bearing walls are made of calcium silicate masonry in which the external wall has three layers—a 30 cm inner load-bearing layer and a 2–3 cm air gap in between the 12 cm external layer. Calcium silicate bricks were taken from various places located in the east- and north-oriented façades to cover a wide range of specimens exposed to climatic loads. Bricks were taken from multiple façade areas having rather harsh wind-driven rain loads such as on building corners, below windows, at different heights, etc., but not close to the eaves. To test the silicate bricks, the bricks were removed as a whole by cutting the brick loose along the mortar. A segment of the wall, consisting of the bricks and mortar, was removed to make the silicate aggregate.

“Ku” was a cement slurry basin, constructed in 1960 (coordinates 59.497983, 26.530396, ca. 3 km south of the Gulf of Finland) (Figure 1 (middle and right)). The thickness of the massive masonry was 38 cm without air gaps. The building was demolished with heavy equipment hydraulic hammers. The masonry segments were transported to Tallinn University of Technology and broken into smaller pieces with impact hammers. The stones were selected at random. 

### 2.2. Laboratory Studies

Laboratory experiments for bricks and silicate aggregate (results described in Section 2.2.1) as well as concrete and mortar (results described in Section 3) were carried out in the Research and Testing Laboratory of Building Materials at Tallinn University of Technology, accredited by the Estonian Accreditation Centre (accreditation No. L004) and the study laboratory at Tallinn University of Technology. Coarse (# 8–16 mm and # 4–8 mm) and fine (# 0–4 mm) aggregate was prepared from recycled silica brick masonry and its mortar mixture by being crushed in a laboratory jaw crusher. The aggregates were used to produce normal concrete by partial replacement of the primary natural aggregate—natural limestone as coarse and silica sand as fine aggregate. The methods for sample preparation, mixture proportion, curing, and testing were chosen according to relevant valid standards listed in the paper. By choosing the standardized methodologies, the repeatability during test period was guaranteed. It is also possible to reproduce mixes according to presented values.

#### 2.2.1. Properties of Calcium Silicate Bricks Used to Produce Concrete and Mortar

The production of calcium silicate masonry bricks in Estonia started in 1910. It can be seen from Table 1 that the compressive strengths of the tested silicate bricks significantly exceed those reported in both historical and modern sources. The specificity of silicate brick production lies in the variability of the compacting of the raw brick before it is autoclaved. Consequently, the density and compressive strength of the hardened masonry also vary.

The compressive strengths of bricks were tested according to European masonry units standard EN 772-1 [21] and the density according to EN 772-13 [22]. The mean compressive strength of silicate brick was 41.1 N/mm^2^ (standard deviation 6.8), and the mean density was 1970 kg/m^3^ (standard deviation 47). The dispersion of the strength will also affect the consumption of cement in the production of concrete. This is because the strength reserve of concrete is related to the standard deviation of compressive strength of concrete according to European concrete standard EN 206 [23]. As the standard deviation of the compressive strength of concrete increases, the required mean compressive strength of concrete increases according to Equation (1) [23].

(1) $$f_{cm}\geq \left(f_{ck}+1.48\cdot \sigma \right)$$

where:

*f_cm_*—mean compressive strength of concrete;
*f_ck_*—characteristic compressive strength of concrete;
*σ*—standard deviation of a population of concrete’s compressive strength.

For the production of aggregates from silicate brick masonry, the water absorption of the aggregate is also an important parameter. Water absorption determines the frost resistance of the concrete and the rate of loss of consistency. The water absorption of silicate bricks was determined according to EN 771-2 [24], and the mean result was 8.1%.

In cold climate conditions such as those in Estonia, the durability of building materials is important for their freeze–thaw resistance. The frost resistance of silicate bricks was determined according to EN 772-18 [25]. The frost resistance of the silicate bricks was determined by holding the bricks for 50 cycles alternately in water at +20 °C and in a cold chamber at −15 °C. The compressive strengths of the bricks subjected to the 50 freezing cycles were compared with the (reference) compressive strengths of the silicate bricks taken from the same locations (uncycled). The compressive strength loss R_c_ was calculated as defined in EVS-EN 772-1 [21]. Silicate bricks originally subjected to outdoor conditions and bricks used in indoor conditions were tested separately. 

The strength and durability properties of calcium silicate masonry bricks are presented in Table 2. 

The loss of strength of the outdoor bricks was R_c_ = 9.4%, and the loss of strength of the indoor bricks was R_c_ = 18.6%. The differences in the results obtained may be due to the variability of the strengths of the silicate bricks. However, it can be concluded from the data that, in time, the outdoor environment does not lead to a decrease in the frost resistance of silicate bricks, and that there is no need to separate bricks that have already undergone freezing cycles from uncycled bricks for the production of concrete.

In general, it can be concluded that despite the high water absorption of the silicate bricks, there was no visible damage during and after freeze–thaw cycling. The compressive strengths of all the silicate bricks tested were in accordance with the compressive strength requirements for new silicate bricks.

#### 2.2.2. Properties of Silicate Aggregate

**Figure 2:**
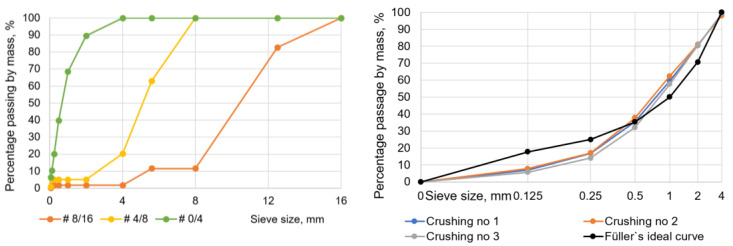
Grading of the aggregate for concrete (**left**). Grading of the aggregate for mortar (**right**).

A laboratory jaw crusher was used to crush the silicate bricks. Together with the silicate bricks, the cement-based masonry mortar attached to them was also crushed. The crushed silicate was sieved into three fractions, and the grading of the aggregate is shown in Figure 2. The grading of crushed calcium silicate bricks was determined for three different batches from the jaw crusher. From the sieve curves, the variation of the grain size is not high.

Looking at the grain size composition of the crushed calcium silicate sand (CCSS) from the jaw crusher in Figure 2 (right), it can be seen that in the range of 0.5–4 mm, the sieve curve follows a linear distribution close to the Füller ideal curve. The lower end of the curve is no longer linear, but is also close to the Füller ideal curve. The grain size composition of the silica sand also shows a dominance of larger particles, with grains in the 0.5–4 mm range accounting for more than 60% of the test samples.

In the context of crushing, it is important to bear in mind that crushing in the laboratory gives a different grading than on site, due to the parameters of the crushers, the crusher technology, and the water content of the bricks. At the site, crushing is carried out first with hydraulic hammers and then with jaw or rotor crushers, with water content as at the time of demolition. When crushing wet material, a higher percentage of fines is produced than when crushing dry material due to the softening factor.

**Figure 3:**
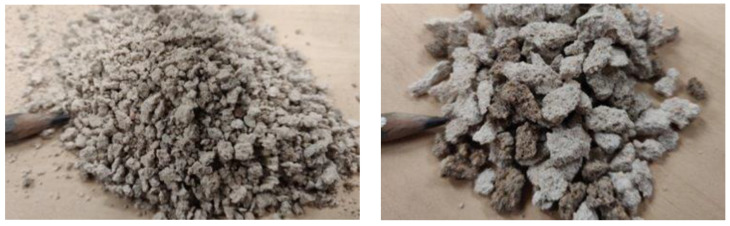
Crushed calcium silicate brick masonry # 0/4 mm (**left**) and # 4/8 mm (**right**). Lighter coarse fractions (silicate brick) and darker (cement mortar) particles are visible.

In addition to the grain size composition of the aggregate produced by the crushing of the silicate brick, the following laboratory tests were carried out and the results are presented in Table 3 and aggregates are shown in Figure 3:

- Bulk density EN 1097-3 [26];
- Water absorption EN 1097-6 [27];
- Resistance to fragmentation (Los Angeles coefficient) EN 1097-2 [28];
- Freeze/thaw resistance EN 1367-1 [29].

The specified properties of silicate aggregates—water absorption, crushing resistance, and frost resistance as determined by traditional aggregate test methods—are significantly lower than those of primary aggregate (natural limestone). The possibilities for recycling the aggregates obtained from the crushing of silicate masonry are therefore limited. Table F.1 in EN 206 [23] gives the recommended characteristics for raw materials for the production of frost-resistant concrete. Table F.1 states that frost-resistant aggregates with low water absorption should be used to produce frost-resistant concrete. Due to the high water absorption of silicate aggregate and low frost resistance (Table 2), the frost resistance criteria of the standard are not met. Still, use of this material can be considered at low static loads and non-freezing conditions.

#### 2.2.3. Components of Mortar

The mortars were prepared in advance as dry mixes. The dry mixes were prepared from the materials listed in Table 4 below. The mixtures were mixed in a ratio of 1/3, one part cement and three parts sand. Plasticizer was added at 0.05% and 0.2% by weight of cement. In addition, a series of mixtures were made with 0.08% plasticizer and 0.05% water retention additive to study mixtures like plasters. The recycled crushed calcium silicate sand is abbreviated “CCSS”.

Dry mixes of different compositions were prepared to investigate the potential uses of calcium silicate bricks. In the mixtures, natural sand was substituted by crushed CCSS at a rate of 25%. To adjust the properties of the mortars, varying amounts of plasticizer and water retention additives were added. The first mix CCSS_0_ is a reference mix of natural sand without additives. The ratios of the dry mixes prepared are given in Table 5.

## 3. Results

### 3.1. Properties of Concrete Made of Calcium Silicate Aggregate

The normal concrete was made from CEM I 42.5R cement at 320 kg/m^3^ without chemical additives. A total of 1 + 6 (reference + tested) different aggregate mixes were used to produce concrete with the following proportions in Table 6.

A constant consistency was aimed for across the seven different mixes. The consistency was determined according to EN 12350-2 [30]. The density of the concrete mix was determined according to EN 12350-6 [31]. The compressive strength of the concrete was tested according to EN 12390-3 [32]. For testing the frost resistance of the concrete, the surface delamination method used in Estonia, Sweden, and Finland was applied. In Estonia, the method for determining frost resistance is described in national standard EVS 814 [33].

The water demand of the concrete increased due to the higher water absorption of calcium silicate aggregate (~10%) compared to the water absorption of limestone aggregate (~2%). The density of the concrete mix decreased with the increase in the proportion of calcium silicate aggregate in the concrete due to the increase in water content and the lower bulk density of calcium silicate aggregate (~960 kg/m^3^) compared to the bulk density of limestone aggregate (~1380 kg/m^3^).

#### 3.1.1. Compressive Strength

**Figure 4:**
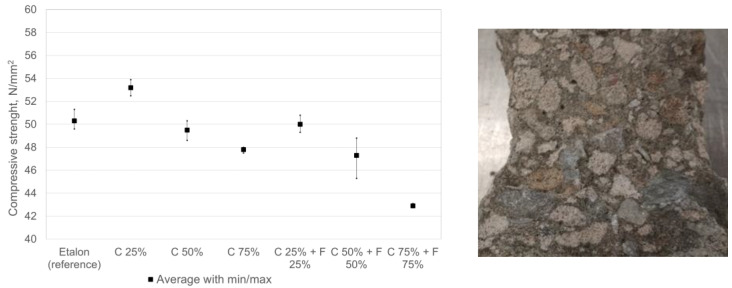
Results of compressive strength of concretes (**left**) and tested specimen (**right**).

The compressive strengths of the concrete were determined at 28 days of age. The concrete specimens were cured for 1 day in the mold and 27 days in water at (20 ± 2) °C. For the average compressive strengths of the concretes, obtained as the average of the three parallel specimens, see Figure 4. The texture of concrete is dense and the grains of limestone as well as calcium silicate aggregate are well bonded to the cement mortar.

The results in the graph show that recycled calcium silica aggregate does not significantly affect the strength properties of concrete but a tendency to lower strengths with higher levels of replaced aggregate can be detected. It is possible to use silicate aggregate as a concrete aggregate, replacing primary concrete aggregates at substitution levels of up to 50%. The increase in compressive strength with up to 25% replacement can be explained by the higher bond strength of the cement due to the rough and porous surface of the CCSS [34], also additional chemical bonding might appear. Further increase (50% and 75%) lowers the compressive strength due to lower density (related to strength) of calcium silicate aggregate (~960 kg/m^3^) compared to that of limestone (~1380 kg/m^3^). More than 50% substitution is not desirable, as it will lead to increased water demand and a stiffer concrete mix structure, which will lead to problems in pouring and compaction due to loss of workability and reduced service life.

#### 3.1.2. Frost Resistance

**Figure 5:**
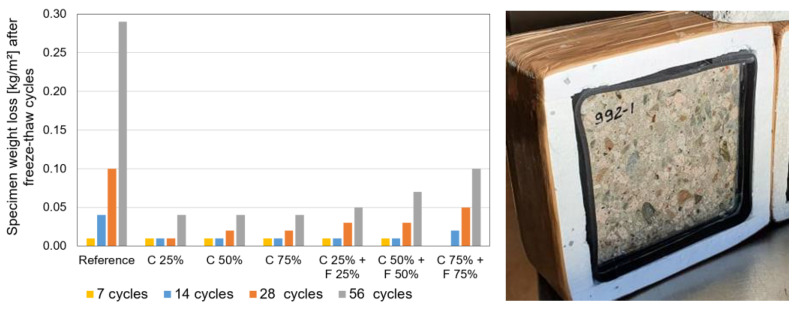
Results of frost resistance of concrete with various amount of replaced coarse (C) and fine (F) aggregate (**left**) and a picture of the freeze-thaw specimen (**right**).

Frost resistance tests of concrete were carried out in accordance with EVS 814 [33]. Freezing and thawing of the test specimens was carried out according to the standard in a climate chamber with forced air circulation. The freezing agent on the surface to be exposed to the test was a 3 mm thick layer of distilled water. The duration of one freeze-thaw cycle was 24 h. After the number of cycles of 7, 14, 28 and 56, the loss in mass of each test specimen was determined, and the total amount of crushed material, Σ M (g), and the total loss in mass per unit area, Σ S [kg/m^2^], were calculated. The average mass loss per unit area of the two test specimens is given in Figure 5.

Although the average mass loss of recycled calcium silica aggregates (22–24%) in the frost resistance test according to EN 1367-1 was ~10 times higher than the corresponding property of primary limestone aggregates (2.2% in Table 2), the frost resistance tests of both whole silicate bricks (through comparison of compressive strength) and concrete made of silicate aggregates show that the use of silicate aggregates as an aggregate for concrete does not significantly impair the frost resistance of concrete. The values given in Figure 5 can be compared with the compliance criteria of EVS 814 [33], which are also given after 56 cycles with values ranging from 0.10 to 1.0 kg/m^2^ (depending on the frost resistance class and service life). It can be concluded that all concretes made with partial replacement of aggregates can be used in EN 206 [23] environmental class XF1 and XF3 (vertical and horizontal concrete surfaces exposed to rain and freezing). Concrete made of secondary (recycled calcium silicate brick masonry) aggregate had lower mass loss compared to the reference concrete made with conventional primary aggregates limestone and sand.

Experimental results on calcium silicate aggregate concretes show that the partial use of calcium silicate (both coarse and fine) aggregate from demolished buildings is realistic and very promising circular options for the future circular construction even in frost resistant concrete.

### 3.2. Properties of Mortar Made of Calcium Silicate Aggregate

Table 4 shows that replacing natural sand with lower-density recycled crushed calcium silicate sand (CCSS) also reduces the bulk density of the aggregate, which is probably going to affect the density and strength of the hardened mortar.

**Figure 6:**
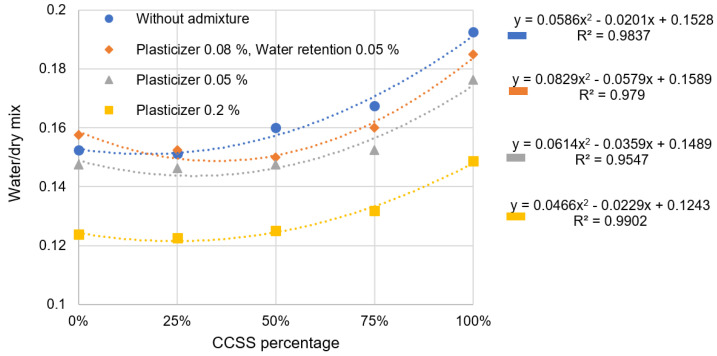
Variation in water demand due to quantities of CCSS and additives.

Figure 6 shows a clear trend that the more CCSS used in a mixture, the higher its water demand. This trendline can be seen in the water demand of each mixture and is consistent with the number of admixtures. Based on dry mixes without additives, the water demand of a 100% CCSS blend is about 21% higher than that of a 0% CCSS mix. This means that a mixture with only CCSS requires 1/5 times more water than a mixture with natural sand. The same result was obtained by Huang [35], Ceylan [36], Omur [37], and Fang [38], who recycled different types of crushed aggregates, which were porous and highly water-absorbent.

Comparing 0% and 25% CCSS mixes, 0% CCSS mixes have a slightly higher water demand than 25% CCSS mixes—about 1%, which is statistically irrelevant. The reduction in water demand is more pronounced in mixtures with 0.08% plasticizer and 0.05% water retention additive. Naturally, the water demand decreases with the plasticizer. It is remarkable that the effect increases with higher CCSS amounts.

The increase in water demand can be attributed to a number of different factors. Several different factors can be expected:

- The water absorption of CCSS is several times higher than that of natural silica sand typically used in masonry mortars.
- The CCSS grain is more angular, so more water is needed to reduce its cohesiveness.
- CCSS has a high proportion of fines in its granular composition, which increases the geometrical area of the sand grains and the water demand of the mixture.

**Figure 7:**
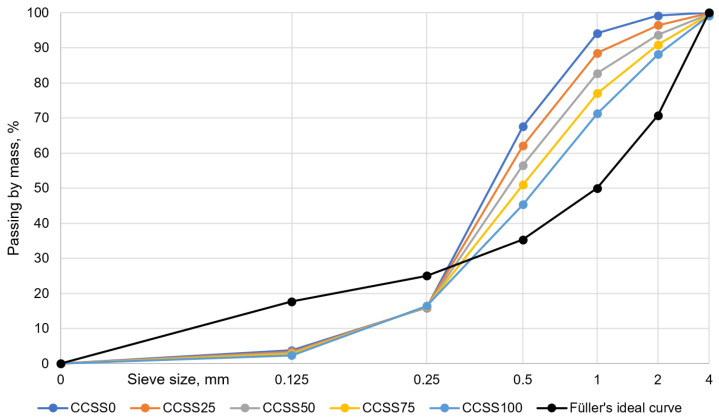
Grading of the aggregate made of mortar.

CCSS was used in mixtures in non-fractionated (all-in) form (shown as “CCSS_0_” in Table 4). Comparing the grain size compositions of natural sand and CCSS, the natural sand has a small amount of the 1–4 mm fraction. In contrast, the CCSS has almost 5 times as many 1–4 mm fractions. In the tested natural sand, ~30% of the weight consists of particles larger than 0.5 mm and more than half of the weight is between 0.25 and 0.5 mm. This is well reflected in the line placement and the difference in slope in the Figure 7. In CCSS, the slope is lower and the sieve curve lower, and in natural sand, the slope is higher and the sieve curve higher.

**Figure 8:**
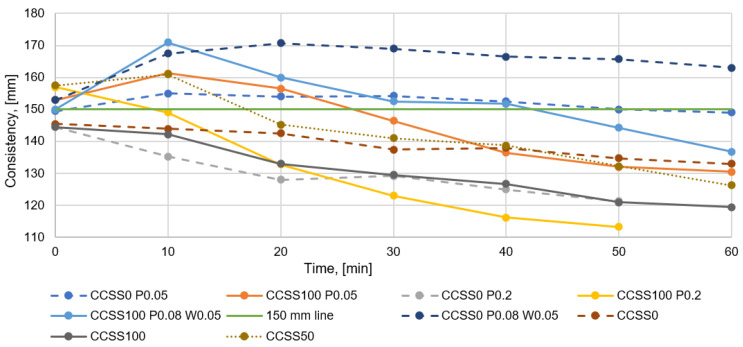
Decrease in consistency of mixtures.

Analyzing Figure 8, all the mixtures tested without admixtures lose consistency over time, which is the expected result. The more CCSS is used in the dry mix, the faster the consistency of the mix disappears. This is due to the high water absorption of CCSS, which absorbs the free water in the mix over time. Exceptionally, if 50% CCSS is used, the consistency will increase in the first 10 min and then decrease in a similar way to other mixes. A small initial increase in consistency is also observed in other tests. The anomaly in the current test may be due to an excessively high initial consistency of the mixture of 158 mm (by default, the study aimed for consistency of 150 mm ± 10 mm). There is a larger amount of water in the mix which cannot all be absorbed by the CCSS and the consistency is higher over time.

Mixtures without admixtures give a good indication of the effect of the CCSS proportion on the consistency of the mix. In order to obtain further confirmation of the issue of whether increasing the proportion of CCSS in a mixture leads to a faster loss of consistency, it is necessary to compare the effect of the proportion of CCSS in mixtures with admixtures on consistency.

Examining the results obtained when using the minimum amount of plasticizer (Figure 8), a trendline can also be seen that the more CCSS is used in the mix, the faster the consistency of the mix is lost. However, some differences can be seen where, for example, with the use of a minimum amount of plasticizer, the consistency of all the mixtures tested increases slightly during the first ten minutes and then starts to decrease. The reason why the consistency may have increased in the first minutes is related to the solubility time of the admixture in the mix. If the admixture does not dissolve quickly enough in the mix, the initial consistency on the flow table may change in later testing. Nevertheless, it can be seen that replacing natural silica sand with CCSS leads to a faster loss of consistency in the mixture.

At the maximum use of the plasticizer (Figure 8), the trend lines are more similar to the mixtures without admixture, where it can be seen that mixes with 75% and 100% CCSS lose their consistency faster. Mixes made with the maximum amount of plasticizer lose consistency more quickly because there is less water in the mix for CCSS to absorb.

Regarding mixes where 0.08% plasticizer and 0.05% water retention admixture were used, a similar trend is also visible for the previously mentioned blends, where the consistency of the mix decreased faster with increasing CCSS content. Overall, it can be seen that the water retention admixture acts as a water retainer in the fresh mix and the CCSS does not absorb excessive amounts of water. The loss of consistency of CCSS can be achieved similar to, and even better than, that of the etalon mix in the algepio mix. Similar to the minimum amount of plasticizer, a trend is also seen where the consistency of the combined mixture increases in the first 10–30 min and then starts to decrease. This may also be due to the solubility time of the admixtures, as observed with the minimum amount of plasticizer.

**Figure 9:**
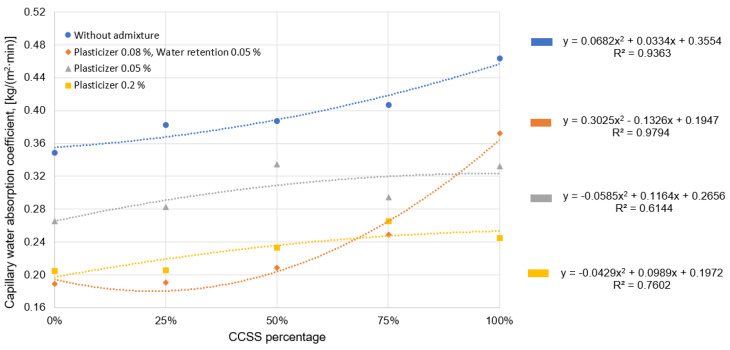
Variation in water absorption due to quantities of CCSS and additives.

Figure 9 shows the general trend that the more CCSS is used in the mixture, the higher the water absorption of the mortar. CCSS with higher water absorption increases the water absorption of mortar. As in the case of high water absorption of CCSS, the slags used in the Omur study [37] had high water absorption, which also increased the water absorption of the hardened mortar. The water absorption in mortar is also increased by voids in the mixture due to large fractions of silica sand. Omur [37] came to a similar conclusion in his study, where he found that more angular aggregate grains create more voids in the mix and increase the water absorption of the hardened mortar.

Comparing mortar with 0% silica sand to mortar with 100% CCSS, the difference in water absorption is almost 25%. The difference in water absorption decreases with increasing the amount of plasticizer, but is still 16% higher for 100% CCSS mortar than for 0% CCSS mortar.

**Figure 10:**
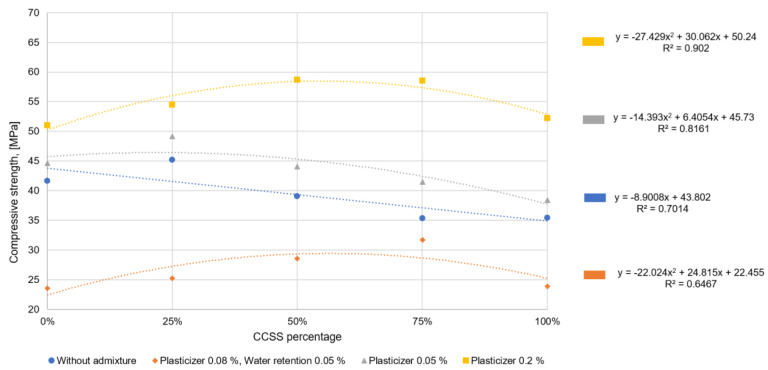
Variation in compressive strength due to quantities of CCSS and additives.

There is a significant difference in water absorption between 0.08% plasticizer and 0.05% water retention additive, where the difference in water absorption is almost 50% for a mixture made with natural sand only and a mixture made with 100% CCSS. The large difference may be since the 100% CCSS mix did not compact sufficiently during molding (Figure 10).

The addition of a small amount of CCSS does not increase the water demand and does not significantly reduce the mortar density. The compressive strength, however, increases with all additive dosages. This is probably related to pozzolanic reactions. At higher CCSS dosages, the density of the hardened mortar decreases significantly and the water/solids factor increases, which leads to a decrease in strength. Similar results have been obtained in studies [39,40] where crushed and ground glass has been used as a recycled aggregate.

## 4. Discussion

Results from using the silicate brick masonry as recycled aggregate in concrete have been presented. Results show good performance in terms of compressive strength, durability and workability. Although water absorption and strength of recycled aggregate itself does not fulfill the requirements in standards, replacing the natural aggregate up to 50% did not degrade the properties of new concrete. If more than 50% of calcium silicate aggregate is used, the stiffness of mixes increases, working time decreases. Also, strength and durability properties become lower.

Frost resistance better than the reference concrete might be associated with the density of the aggregate. A lower density, higher (closed) porosity aggregate may act as a reserve buffer in the concrete as a reducer of hydraulic pressure due to water and ice [19,41].

Direct comparison with previous studies is complicated because no earlier publication about concrete made of recycled calcium silicate brick aggregate was found. There are many studies where concrete, ceramic brick or other mineral CDW is used for aggregate [42]. Ceramic bricks crushed into a powder with a particle size 25–100 μm exhibit a positive impact on the compressive strength of both mortars and concretes with a maximum cement replacement level of 10–20% [43]. Replacing natural aggregate with recycled concrete has shown acceptable performance, but as expected, the results depend on the aggregate. For instance, compressive strength does not decrease more than 3.5 MPa (i.e., 8.8%) for class 40 MPa concrete and not more than 3.1 MPa (i.e., 5.2%) for class 60 MPa concrete [20,44]. The results of concrete with recycled clay brick and tile indicate a slightly higher slump, lower unit weight, and higher air content [21,45]. Compressive strength and elastic modulus of concrete with clay brick and tile aggregate decreased by 15–25% and 25%, respectively, against the reference material [21,45]. In general, the properties of concrete containing recycled concrete aggregate worsen because of old cement [22,46]. Recycled cement causes higher porosity, which leads to lower density and higher water absorption of concrete. On the other hand, recycled aggregate might improve the wear resistance. The circularity is limited—a concrete made of recycled concrete can made twice, but after that, the properties and performance of concrete drop rapidly [22]. When it comes to mortars, expected requirements, properties and behavior (including the effect of admixtures) depends on its use conditions and application technology, e.g., 3D printing [47].

Concerning future research, moisture content (possible additional wetting) of recycled aggregate should be studied, as pre-wetted recycled aggregate decreases the need for water. A second factor to be considered is the loss of workability over time, as fresh concrete containing recycled aggregate absorbs some of the water needed for workability. It cannot be directly calculated from the water absorption comparison between primary limestone aggregate against recycled silicate brick aggregate because it is uncertain how much water is sucked into the aggregate, how much reacts with cement during the hydration, and how much is left for workability. Therefore, additional practical testing for loss of workability over time (e.g., after 1/6, 1/3, 1/2, 1, 2, 3 and 4 h after mixing the fresh concrete) is needed to maintain the required properties before the concrete is poured on site. Different treatments of aggregates could be a solution to reduce the water absorption (up to 65%) and increase levels of compressive strength (up to 37%), and improve workability [23,48]. As all the tests were carried out without any chemical admixture in the concrete, future tests could characterize the effect of different chemical admixtures (e.g., superplasticizers, water retention increasing admixture and air entraining admixture) on the strength and durability of the concrete. There is evidence that chemical admixtures such as polycarboxylate ester with limestone cement improves the physical–mechanical and viscosity properties of concrete [49], and chemical admixtures can be used to create synergy interaction in rheological properties of foamed cement pastes in lightweight concrete from industrial technogenic waste [50]—therefore, future research should be aimed at balancing out possible negative impacts (such as higher water absorption) of using recycled aggregate.

## 5. Conclusions

In conclusion, the study demonstrates the potential of recycled calcium silicate brick masonry as a viable alternative aggregate in concrete and mortar production. The properties of concrete aggregates made of calcium silicate brick masonry, tested as concrete aggregates, do not meet many of the criteria set out in EN 12620 [51] for concrete aggregates, but, as constituents of concrete, their performance improves significantly. The results indicate that incorporating recycled aggregate up to 50% does not significantly compromise concrete properties, including compressive strength and durability. Depending on the rate of replacement, it is possible to produce concrete with a medium strength class C30/37 from recycled calcium silicate aggregate. Although the requirements for aggregates in the concrete standard EN 206 [23] state that frost-resistant aggregates should be used to produce frost-resistant concrete, our tests have shown that it is possible to produce frost-resistant concrete from silicate aggregate. However, challenges such as increased water demand and loss of workability over time are observed with higher proportions of recycled aggregate, highlighting the need for further research to address these issues. Despite these challenges, the findings suggest that recycled calcium silicate brick aggregate holds promise as a sustainable solution for reducing the need for primary concrete aggregate and upcycling of construction and demolition waste. Results of current research could be practically applied on-site in post-war and post-disaster countries, where calcium silicate brick masonry is available.

## Figures and Tables

**Table 1:** Properties of calcium silicate bricks through the ages.

Origin of the Bricks	Density, kg/m^3^	Compressive Strength, N/mm^2^
Calcium silicate bricks in the Czech Republic [18]	1661–1874	32 (average)
Calcium silicate bricks in Estonia 1960-s [19]	1920–2080	43 (average)
Calcium silicate bricks in Estonia 1970-s [19]	1860–2010	36 (average)
Calcium silicate bricks in Estonia 2010–2020 [20]	1850–1950	≥25

**Table 2:** Frost resistance of silicate masonry units.

The Location of Brick	Compressive Strength of Uncycled Bricks, N/mm^2^		Compressive Strength of Bricks after 50 Freeze–Thaw Cycles, N/mm^2^	
	Individual Result	Mean	Individual Result	Mean
From external wall	62.1	60	46.6	54
	64.1		52.9	
	61.7		56.2	
	50.3		60.4	
From internal wall	62.7	57	37.5	46
	62.1		46.6	
	64.1		52.9	
	61.7		56.2	

**Table 3:** Properties of aggregates made of crushed calcium silicate brick.

Properties	Unit	Aggregate Size						
		Silicate Brick Aggregate for					Natural Sand	Limestone Aggregate
		Concrete				Mortar		
		# 0/4	# 4/8	# 8/16	# 10/14	# 0/4	# 0/4	# 4/16
Bulk density	g/cm^3^	1.27	0.90	0.96		1.37	1.52	1.38
Water absorption	%	4.7	10.4	9.8		3.9	0.1	2.4
Los Angeles coefficient	-				76.1			30
Freeze/thaw resistance	%		21.8	23.5				2.2

**Table 4:** Raw materials of mortars.

Material	Producer	Descripiton
Cement	Heidelberg Materials Kunda AS, Lääne-Viru maakond, Estonia	CEM II/A-M(T-L) 42.5 R
Plasticizer	Imerys S.A, Paris, France	Peramin^®^ CONPAC 700
Water retention additive	Nouryon HQ, Amsterdam, The Netherlands	Bermocoll M 30
Natural sand	AS Silikaat, Tallinn, Estonia	Sand quarry “Saku” # 0/4
CCSS	Authors	# 0/4

**Table 5:** Mortar composition proportions.

Sample Identification	Primary Aggregate (Sand) [%]	Recycled Aggregate (CCSS) [%]	Plasticizer [%]	Water Retention Additive [%]	Bulk Density of Sand [kg/m^3^]	Water Demand, W/DM Ratio [-]
CCSS_0_	100	0	-	-	1515	0.153
CCSS_0_P_0.05_			0.05	-		0.148
CCSS_0_P_0.2_			0.2	-		0.124
CCSS_0_P_0.08_W_0.05_			0.08	0.05		0.158
CCSS_25_	75	25	-	-	1503	0.151
CCSS_25_P_0.05_			0.05	-		0.146
CCSS_25_P_0.2_			0.2	-		0.123
CCSS_25_P_0.08_W_0.05_			0.08	0.05		0.153
CCSS_50_	50	50	-	-	1472	0.160
CCSS_50_P_0.05_			0.05	-		0.148
CCSS_50_P_0.2_			0.2	-		0.125
CCSS5_0_P_0.08_W_0.05_			0.08	0.05		0.150
CCSS_75_	25	75	-	-	1418	0.168
CCSS_75_P_0.05_			0.05	-		0.153
CCSS_75_P_0.2_			0.2	-		0.132
CCSS_75_P_0.08_W_0.05_			0.2	0.3		0.160
CCSS_100_	0	100	-	-	1370	0.193
CCSS_100_P_0.05_			0.05	-		0.176
CCSS_100_P_0.2_			0.2	-		0.149
CCSS_100_P_0.08_W_0.05_			0.2	0.3		0.185

**Table 6:** Aggregate proportions and properties of fresh concrete.

Sample Identification	Primary Aggregate		Recycled Aggregate		w/c Ratio	Properties of Fresh Concrete	
	Coarse	Fine	Coarse	Fine		Density, kg/m^3^	Slump, cm
Reference	100%	100%	-	-	0.61	2440	4.0
C 25%	75%	100%	25%	-	0.63	2420	3.0
C 50%	50%	100%	50%	-	0.65	2360	3.5
C 75%	25%	100%	75%	-	0.66	2290	1.0
C 25% + F 25%	75%	75%	25%	25%	0.65	2390	1.5
C 50% + F 50%	50%	50%	50%	50%	0.69	2330	1.0
C 75% + F 75%	25%	25%	75%	75%	0.81	2240	1.5

## Data Availability

Data will be available upon request.
